# Supplementary material for: E proteins control the development of NKγδT cells through their invariant T cell receptor
Source: Nat Commun. 2024 Jun 13;15:5078. doi: 10.1038/s41467-024-49496-3 (PMC11176164; doi:10.1038/s41467-024-49496-3)
Supplement: Supplementary file 1 — Supplementary Information [file 41467_2024_49496_MOESM1_ESM.pdf]

SUPPLEMENTARY FIGURES

E proteins control NKγδT development through both generation and function of their stereotypic TCR

Ariana Mihai, Sang-Yun Lee, Susan Shinton, Mitchell I. Parker, Alejandra V. Contreras, Baojun Zhang, Michele Rhodes, Roland L. Dunbrack, Juan-Carlos Zúñiga-Pflücker, Maria Ciofani, Yuan Zhuang, David L. Wiest.

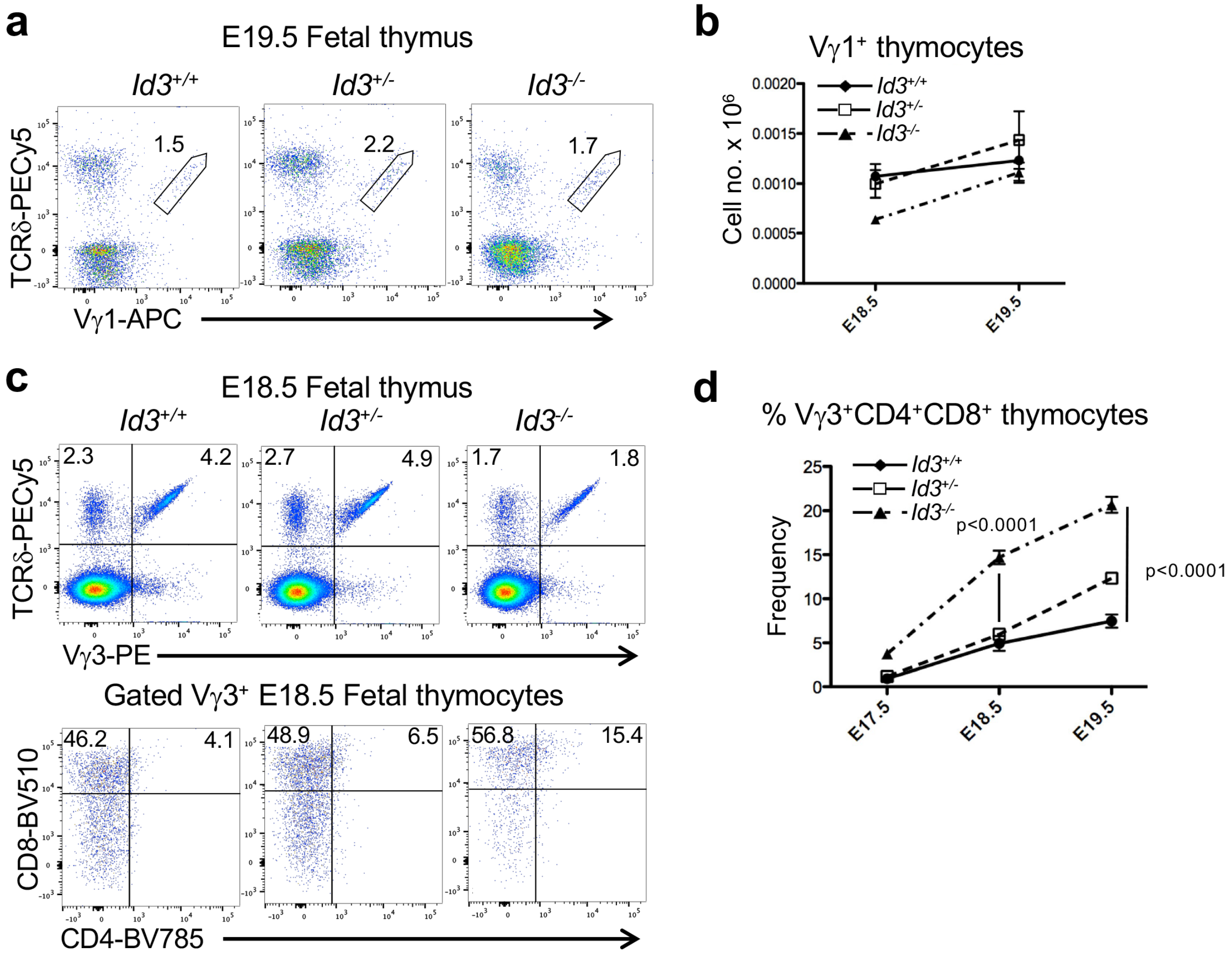

Supplementary Fig. 1. Impact of Id3-deficiency on fetal development of Vγ1.1+ γδ T cells

(a,b) (a) Representative flow cytometry plots depicting the frequency of Vγ1.1+ γδ T cells in E19.5 fetal thymic organ culture (FTOC) of fetal thymocytes from *Id3+/+, Id3+/-,* and *Id3-/-* mice. **(b)** The mean ± SD of the absolute number of Vγ1.1+ γδ T cells per lobe at E18.5 and E19.5 is depicted graphically. **(c,d)** Diversion of Vγ3+ thymocytes γδ T to the αβ fate was assessed by gating on Vγ3+ (upper panel) and displaying CD4 and CD8 expression (lower panel). The mean ± SD of the frequency of Vγ3+ cells diverted to the αβ T cell fate, as indicated by becoming CD4+8+ double positive thymocytes, is depicted graphically for fetal thymocytes at E17.5, E18.5, and E19.5 of FTOC for mice of the indicated genotypes. Representative flow cytometry plots are displayed. 4 thymic lobes were analyzed per genotype at each time point. All data represent at least 3 independent experiments. Statistical analysis: two-way ANOVA with correction for multiple comparison using Tukey's post hoc testing. p values are indicated on the Figure.

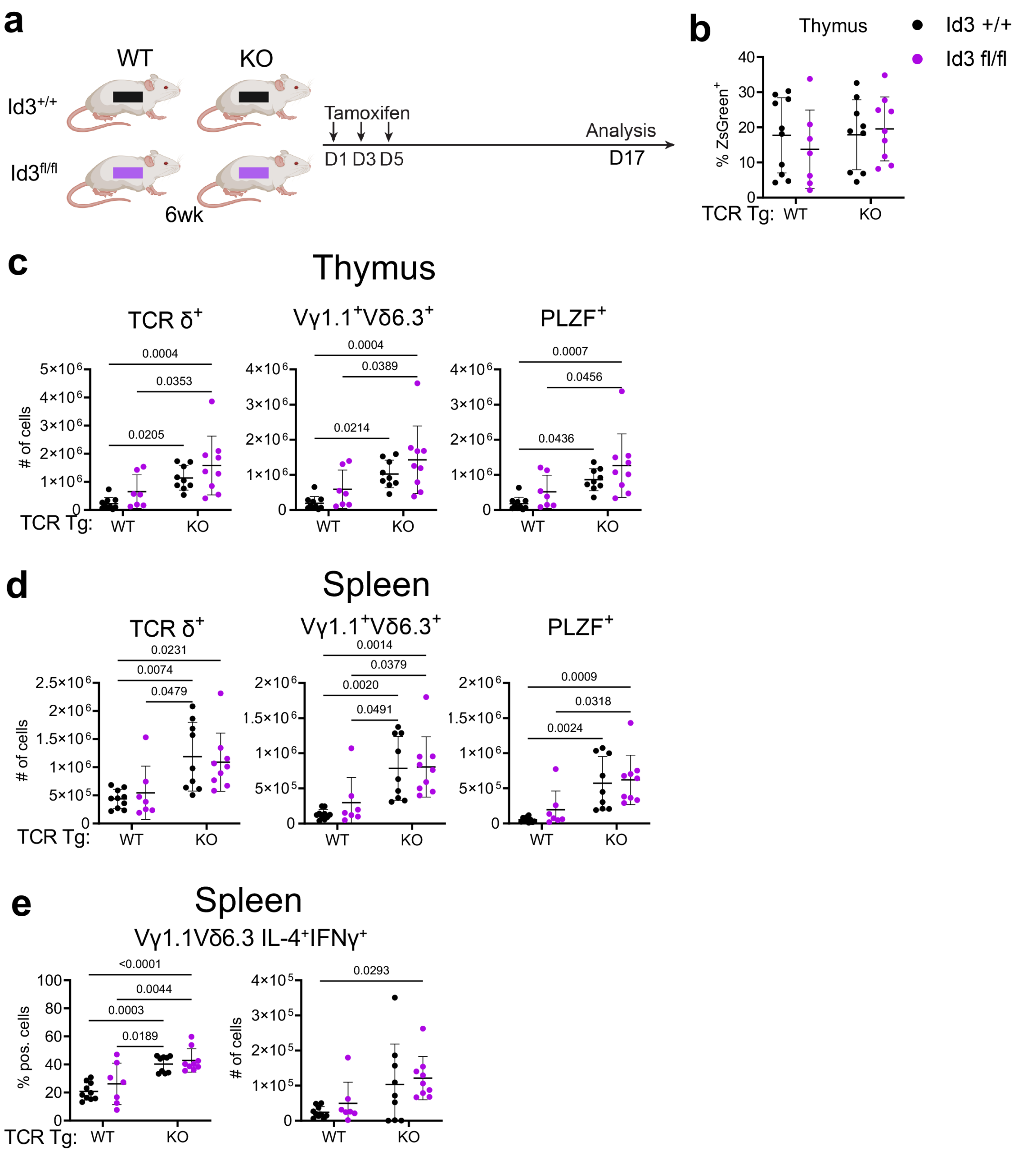

Supplementary Fig. 2. Impact of conditional, TCRd-Cre mediated ablation of *Id3* on development of Vγ1.1Vδ6.3 TCR expressing NKγδT cells.
(a) Diagram of *in vivo* experimental design constructed using BioRender. Tamoxifen was administered to 6-week-old mice on days 1, 3, and 5, and analysis was performed on day 17. **(b)** Graphic depiction of the mean ± S.D. of the frequency of ZsGreen^{+} thymocytes is overlayed on the scatter grams.. **(c,d)** Mean ± S.D. Number of thymic **(c)** or splenic **(d)** TCRδ^{+}, Vγ1.1^{+}Vδ6.3^{+}, and PLZF^{+} Vγ1.1^{+}Vδ6.3^{+} cells is depicted graphically with each symbol representing a single mouse. **(e)** Graphic representation of the frequency and number of IL-4 and IFNγ co-producing Vγ1.1^{+}Vδ6.3^{+} splenocytes following PMA/Ionomycin stimulation, with each symbol representing a single mouse. *TCR^{WT}Id3$^{+/+}$* (n = 10), *TCR^{WT}Id3$^{fl/fl}$* (n = 7), *TCR^{KO}Id3$^{+/+}$* (n = 9), and *TCR^{KO}Id3$^{fl/fl}$* (n = 9). Data were pooled from 3 independent experiments and are plotted as mean±SD. Statistical analysis: two-way ANOVA with correction for multiple comparison using Tukey's post hoc testing p values are indicated on the Figure.

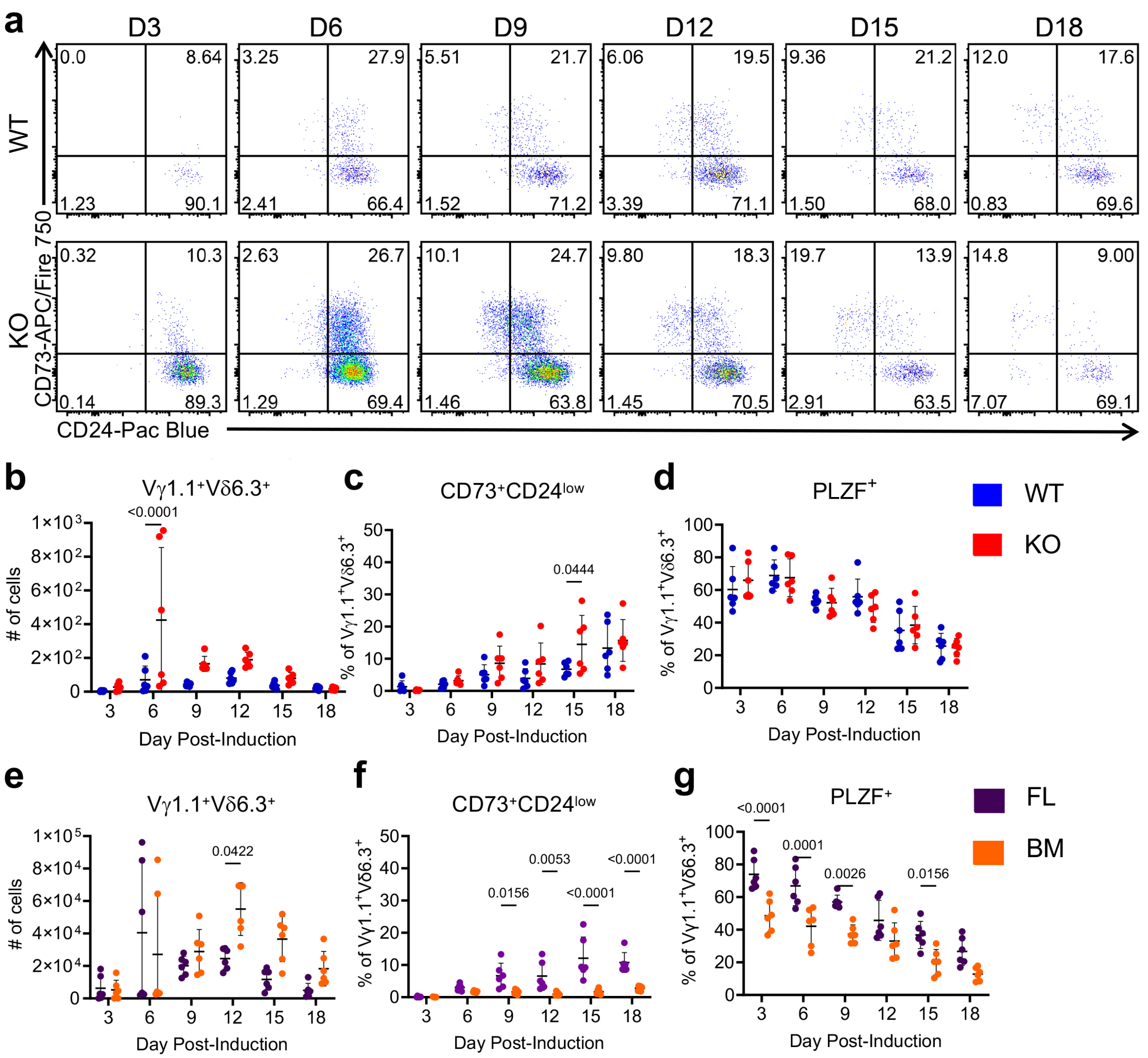

Supplementary Fig. 3. Capacity of Vγ1.1Vδ6.3 TCR complexes to instruct the NKγδT cell fate in vitro.

(a) Representative flow cytometry plots of FL derived Vγ1.1Vδ6.3 T lymphocyte maturation in culture displayed as CD73 vs CD24. The WT and KO Tg TCRs were induced in FL derived precursors cultured in T cell polarizing conditions. **(b-d)** Graphic depiction of the **(b)** absolute numbers Vγ1.1Vδ6.3 expressing cells obtained, **(c)** frequency which were CD73^{+}CD24^{low}, and **(d)** frequency of Vγ1.1Vδ6.3 cells expressing PLZF. **(e-g)** The KO Tg TCR was induced in FL and bone marrow derived precursors cultured in T cell polarizing conditions. Graphic depiction of the **(e)** absolute numbers Vγ1.1Vδ6.3 expressing cells obtained, and the frequencies of these which were **(f)** CD73^{+}CD24^{low} or **(g)** expressed PLZF. Data are pooled from 3 independent experiments (n = 6). Data are plotted as mean±SD. Statistical analysis: two-way ANOVA with correction for multiple comparison using Sidak's multiple comparisons test. p values are indicated on the Figure.

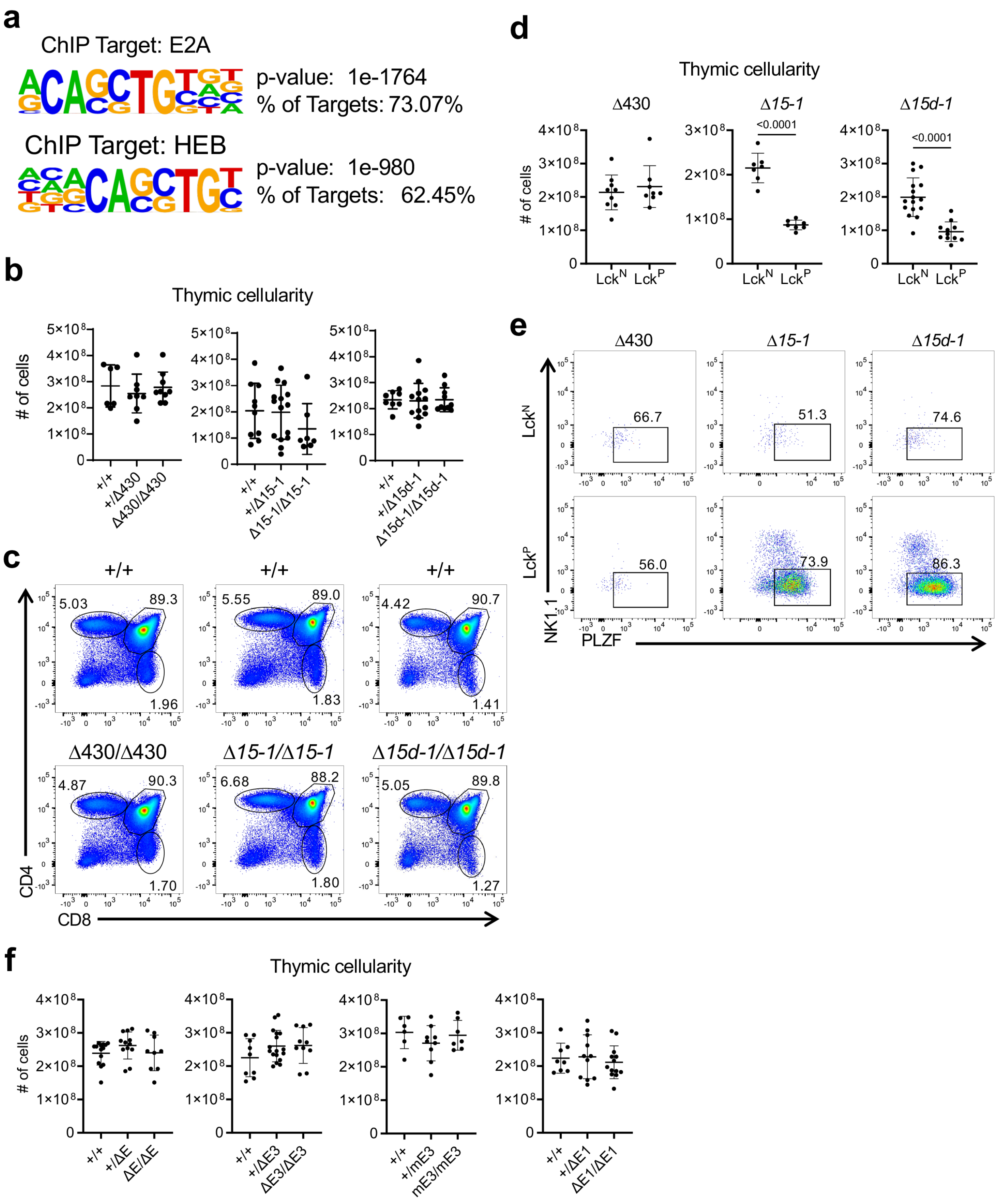

Supplementary Fig. 4. Role of E protein binding in *Trav15* element selection in developing NKγδT cells.

(a) Motif analysis on the E2A and HEB ChIP data was performed with Homer using the findMotifsGenome.pl function (size = 200, masked). The logo, p-value for E2A and HEB binding site enrichment, and the fraction of called binding sites containing a consensus E box are listed. **(b)** Thymic cellularity in *Δ430, Δ15-1*, and *Δ15d-1* mutant mice is depicted graphically with each

symbol representing an individual mouse. All comparisons involved littermates. *Δ15-1: Tcra*$^{+/+}$ (n = 10), *Tcra*$^{+/Δ15-1}$ (n = 15), and *Tcra*$^{Δ15-1/Δ15-1}$ (n = 7); *Δ15d-1: Tcra*$^{+/+}$ (n = 8), *Tcra*$^{+/Δ15d-1}$ (n = 13), and *Tcra*$^{Δ15d-1/Δ15d-1}$ (n = 10); *Δ430: Tcra*$^{+/+}$ (n = 6), *Tcra*$^{+/Δ430}$ (n = 8), and *Tcra*$^{Δ430/Δ430}$ (n = 9). Data were pooled from a minimum of 3 independent experiments and are plotted as mean±SD. Statistical analysis: one-way ANOVA with correction for multiple comparison using Tukey's post hoc testing. Significant differences were not detected. **(c)** Representative flow cytometry plots for subsets defined by CD4 and CD8 expression in *Tcra*$^{+/+}$ and littermate knockout of *Tcra*$^{Δ430/Δ430}$, *Tcra*$^{Δ15-1/Δ15-1}$, or *Tcra*$^{Δ15d-1/Δ15d-1}$ mutant mice. **(d)** Thymic cellularity in Lck-Cre negative (Lck^{N}) Id3 sufficient (*Id3*$^{fl/fl}$) (control), or Lck-Cre (Lck^{P}) mediated Id3 deficient (*Id3*$^{fl/fl}$) *Δ430, Δ15-1,* and *Δ15d-1* mutants. All comparisons to Lck-Cre negative (Lck^{N}) littermates. For *Δ430:* Lck^{N} (n = 9), Lck^{P} (n = 8); *Δ15-1*: Lck^{N} (n = 7), Lck^{P} (n = 7); *Δ15d-1:* Lck^{N} (n = 16) and Lck^{P} (n = 11). Data are pooled from at least 3 independent experiments and plotted as mean±SD. Statistical analysis: Student's t test. **(e)** Representative flow cytometry plots of NK1.1 and PLZF expression in Vγ1^{+} thymocytes from Lck-Cre negative (Lck^{N}) Id3 sufficient (*Id3*$^{fl/fl}$) (control), or Lck-Cre (Lck^{P}) mediated Id3 deficient (*Id3*$^{fl/fl}$) *Δ430, Δ15-1,* and *Δ15d-1* mutants. **(f)** Thymic cellularity in *ΔE, ΔE3, mE3,* and *ΔE1* mutant mice is depicted graphically with each symbol representing an individual mouse. All comparisons involved littermates. *ΔE: Tcra*$^{+/+}$ (n = 14), *Tcra*$^{+/ΔE}$ (n = 12), and *Tcra*$^{ΔE/ΔE}$ (n = 9); *ΔE3: Tcra*$^{+/+}$ (n = 9), *Tcra*$^{+/ΔE3}$ (n = 17), and *Tcra*$^{ΔE3/ΔE3}$ (n = 10); *mE3: Tcra*$^{+/+}$ (n = 6), *Tcra*$^{+/mE3}$ (n = 9), and *Tcra*$^{mE3/mE3}$ (n = 7); *ΔE1: Tcra*$^{+/+}$ (n = 8), *Tcra*$^{+/ΔE1}$ (n = 11), and *Tcra*$^{ΔE1/ΔE1}$ (n = 13). Data were pooled from a minimum of 3 independent experiments and are plotted as mean±SD. Statistical analysis: one-way ANOVA with correction for multiple comparison using Tukey's post hoc testing. Significant differences were not detected. p values are indicated on the Figure.

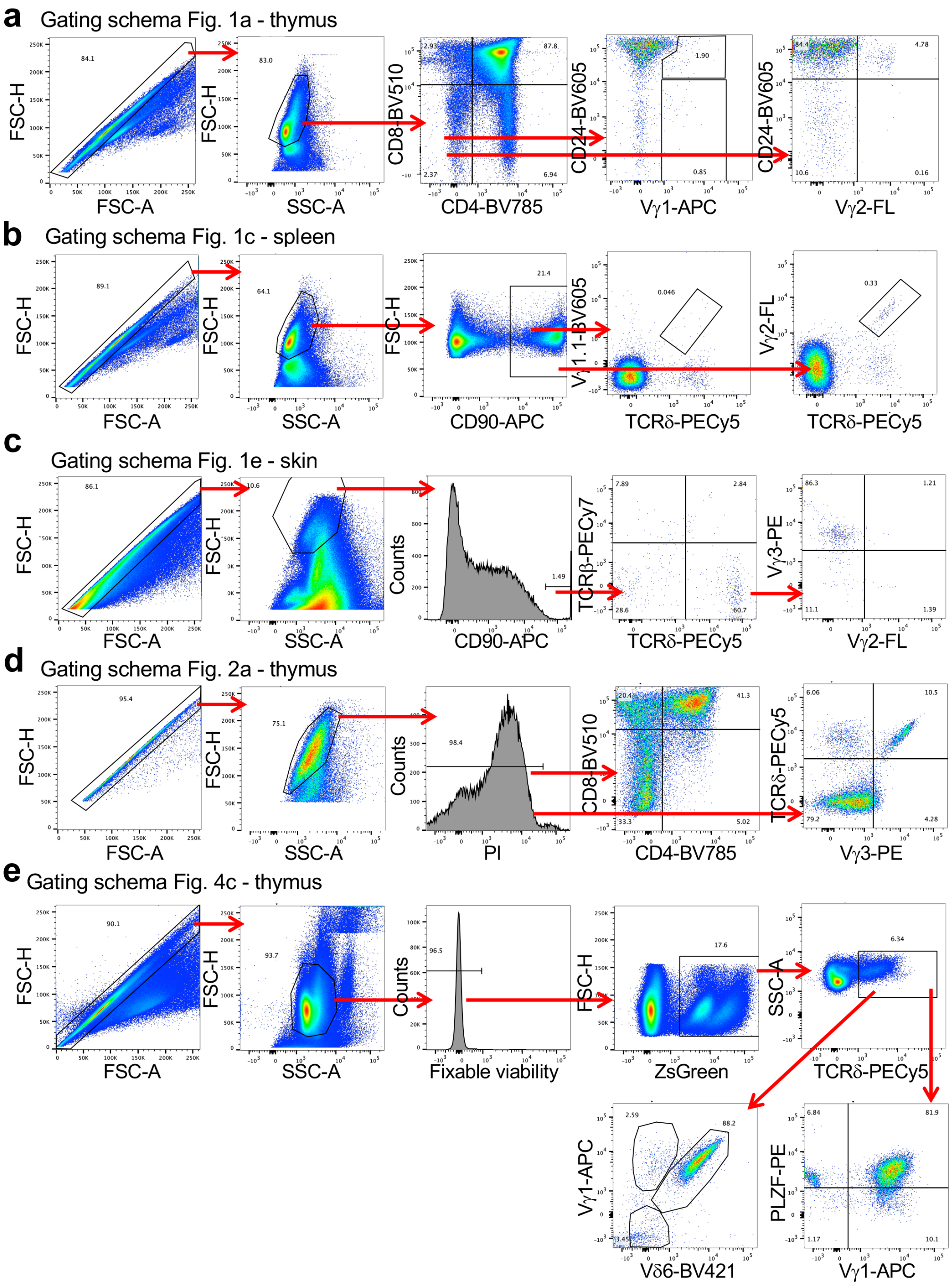

Supplementary Fig. 5. Gating schema for flow cytometry analyses in Figures 1, 2, and 4. Graphical representation for the gating schema are provided for Figures 1a **(a)**, 1c **(b)**, 1e **(c)**, 2a **(d)**, and 4c **(e)**. Red arrows denote the subgates displayed in the subsequent histograms in the schema.

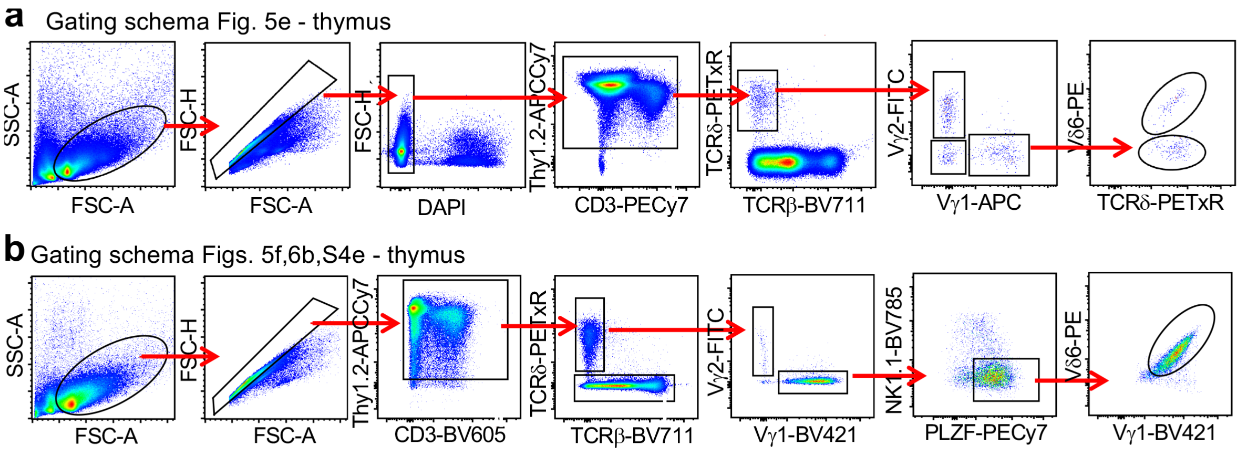

Supplementary Fig. 6. Gating schema for flow cytometry analyses in Figures 5, 6, and S4e. Graphical representation for the gating schema are provided for Figures 5e **(a)**, 5f,6b,S4e **(b)**. Red arrows denote the subgates displayed in the subsequent histograms in the schema.

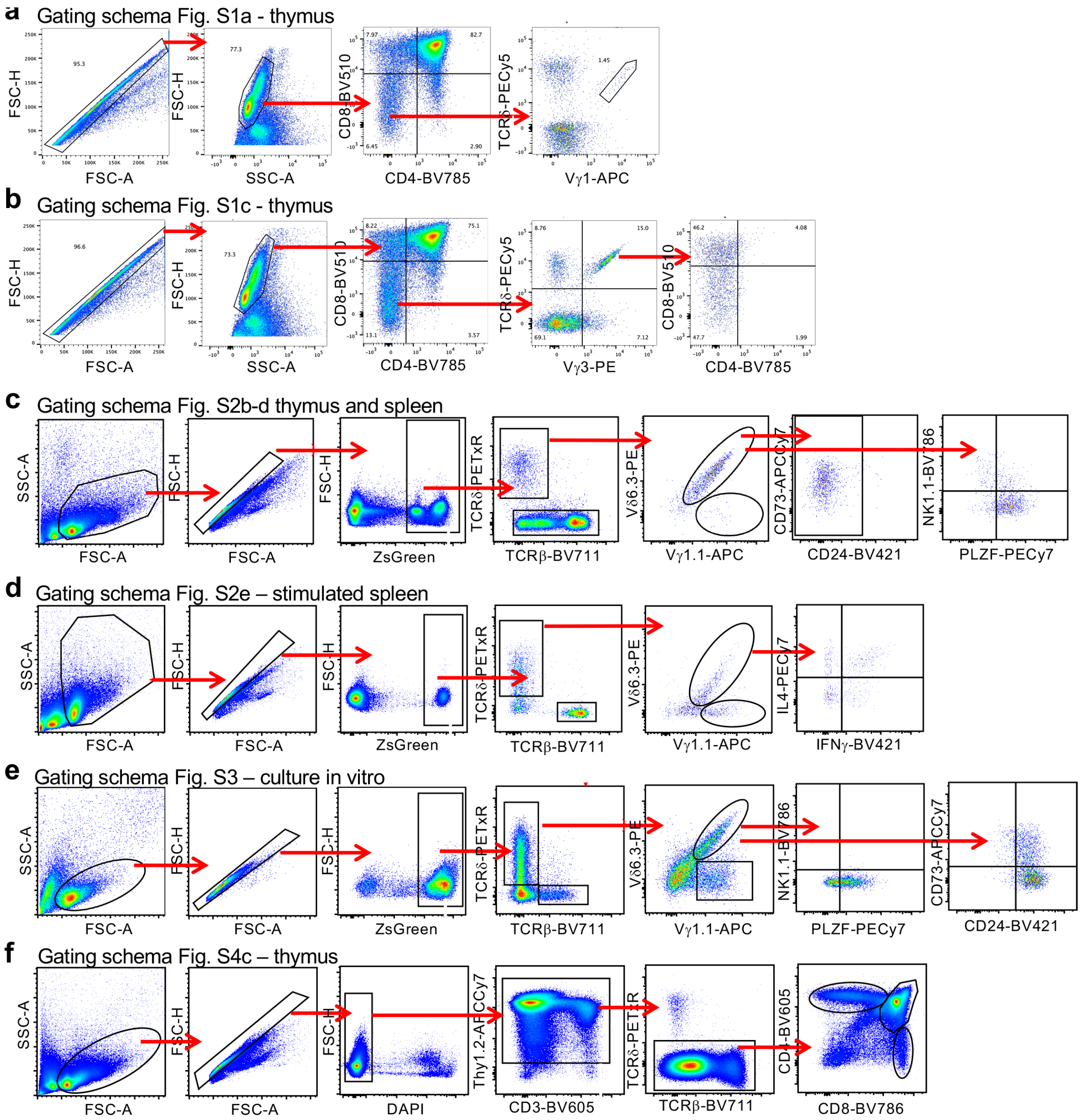

Supplementary Fig. 7. Gating schema for flow cytometry analyses in Figures S1, S2, S3, and S4c. Graphical representation for the gating schema are provided for Figures S1a **(a)**, S1c **(b)**, S2b-d **(c)**, S2e **(d)**, S3 **(e)**, and S4c **(f)**. Red arrows denote the subgates displayed in the subsequent histograms in the schema.
