# Supplementary material for: E proteins control the development of NKγδT cells through their invariant T cell receptor
Source: Nat Commun. 2024 Jun 13;15:5078. doi: 10.1038/s41467-024-49496-3 (PMC11176164; doi:10.1038/s41467-024-49496-3)
Supplement: Supplementary file 3 — Reporting Summary [file 41467_2024_49496_MOESM3_ESM.pdf]

nature portfolio

Corresponding author(s): David L. Wiest

Last updated by author(s): May 24, 2024

Reporting Summary

Nature Portfolio wishes to improve the reproducibility of the work that we publish. This form provides structure for consistency and transparency in reporting. For further information on Nature Portfolio policies, see our Editorial Policies and the Editorial Policy Checklist.

Statistics

For all statistical analyses, confirm that the following items are present in the figure legend, table legend, main text, or Methods section.

n/a	Confirmed	
☐	☒	The exact sample size (*n*) for each experimental group/condition, given as a discrete number and unit of measurement
☐	☒	A statement on whether measurements were taken from distinct samples or whether the same sample was measured repeatedly
☐	☒	The statistical test(s) used AND whether they are one- or two-sided<br>*Only common tests should be described solely by name; describe more complex techniques in the Methods section.*
☒	☐	A description of all covariates tested
☐	☒	A description of any assumptions or corrections, such as tests of normality and adjustment for multiple comparisons
☐	☒	A full description of the statistical parameters including central tendency (e.g. means) or other basic estimates (e.g. regression coefficient) AND variation (e.g. standard deviation) or associated estimates of uncertainty (e.g. confidence intervals)
☒	☐	For null hypothesis testing, the test statistic (e.g. *F*, *t*, *r*) with confidence intervals, effect sizes, degrees of freedom and *P* value noted<br>*Give P values as exact values whenever suitable.*
☒	☐	For Bayesian analysis, information on the choice of priors and Markov chain Monte Carlo settings
☒	☐	For hierarchical and complex designs, identification of the appropriate level for tests and full reporting of outcomes
☒	☐	Estimates of effect sizes (e.g. Cohen's *d*, Pearson's *r*), indicating how they were calculated

Our web collection on statistics for biologists contains articles on many of the points above.

Software and code

Policy information about availability of computer code

Data collection	FACS data was collected using either a BD Fortessa or Symphony flow cytometer.
Data analysis	FlowJo Software (v10,8,2) was used to analyze flow cytometry data. Structure predictions were performed with AlphaFold-Multimer v2.3 (https://www.biorxiv.org/content/10.1101/2021.10.04.463034v2.abstract) as implemented in ColabFold running on local machines. Previously published ChiP-Seq data was reanalyzed by alignment using Bowtie2, peak calling was performed using MACS2, following which data were visualized using Integrative Genomics Viewer.

For manuscripts utilizing custom algorithms or software that are central to the research but not yet described in published literature, software must be made available to editors and reviewers. We strongly encourage code deposition in a community repository (e.g. GitHub). See the Nature Portfolio guidelines for submitting code & software for further information.

Data

Policy information about availability of data

All manuscripts must include a data availability statement. This statement should provide the following information, where applicable:

- Accession codes, unique identifiers, or web links for publicly available datasets
- A description of any restrictions on data availability
- For clinical datasets or third party data, please ensure that the statement adheres to our policy

The resulting ChIP-seq data analyzed here is available in GEO (GEO: GSE162292). All data in the figures are available in the published article or in the online

supplemental material. Unprocessed FCS files are currently available at the FlowLIMS at Fox Chase Cancer Center or by request. After publication primary .fcs files will be deposited in a public database.

Research involving human participants, their data, or biological material

Policy information about studies with human participants or human data. See also policy information about sex, gender (identity/presentation), and sexual orientation and race, ethnicity and racism.

Reporting on sex and gender	Not applicable
Reporting on race, ethnicity, or other socially relevant groupings	Not applicable
Population characteristics	Not applicable
Recruitment	Not applicable
Ethics oversight	Not applicable

Note that full information on the approval of the study protocol must also be provided in the manuscript.

Field-specific reporting

Please select the one below that is the best fit for your research. If you are not sure, read the appropriate sections before making your selection.

☒ Life sciences ☐ Behavioural & social sciences ☐ Ecological, evolutionary & environmental sciences

For a reference copy of the document with all sections, see nature.com/documents/nr-reporting-summary-flat.pdf

Life sciences study design

All studies must disclose on these points even when the disclosure is negative.

Sample size	No sample size calculation was performed. Sample size was determined from experimental aim and the availability of age-matched mice.
Data exclusions	No data were excluded
Replication	Data reproducibility was confirmed by independent experiments.
Randomization	No randomization was used in this study. Covariates were not relevant.
Blinding	Blinding was employed for data collection and then unblinded for genotypic grouping and data analysis.

Reporting for specific materials, systems and methods

We require information from authors about some types of materials, experimental systems and methods used in many studies. Here, indicate whether each material, system or method listed is relevant to your study. If you are not sure if a list item applies to your research, read the appropriate section before selecting a response.

Materials & experimental systems

n/a	Involved in the study	
☐	☒	Antibodies
☐	☐	Eukaryotic cell lines
☒	☐	Palaeontology and archaeology
☐	☒	Animals and other organisms
☒	☐	Clinical data
☒	☐	Dual use research of concern
☒	☐	Plants

Methods

n/a	Involved in the study	
☐	☒	ChIP-seq
☐	☒	Flow cytometry
☐	☐	MRI-based neuroimaging

Antibodies

Antibodies used	Cells were isolated and stained with the following antibodies: anti-CD3 (17A2), anti-CD90.2 (30-H12), anti-CD4 (GK1.5 or RM4-5), anti-CD8 (53-6.7), anti-CD24 (M1/69), anti-CD73 (TY/11.8), anti-CD122 (TM-b1), anti-TCRβ (H57-597), anti-TCRδ (GL3), anti-Vγ1

	(2.11), anti-Vγ2 (UC3-10A6), anti-Vγ3 (536), anti-Vd6.3 (C504.17C), anti-PLZF (9E12), anti-NK1.1 (PK136), anti-IL4 (11B11) and anti-IFNg (XMG1.2).
Validation	All flow cytometry antibodies are commercially available and have been validated by the manufacturer.

Eukaryotic cell lines

Policy information about cell lines and Sex and Gender in Research

Cell line source(s)	Not applicable
Authentication	Not applicable
Mycoplasma contamination	Not applicable
Commonly misidentified lines (See ICLAC register)	Not applicable

Animals and other research organisms

Policy information about studies involving animals; ARRIVE guidelines recommended for reporting animal research, and Sex and Gender in Research

Laboratory animals	TcrdCreER, Id3-/-, Id3fl/fl, and LckCre mice on a C57BL/6 background that were generated previously were employed in this study. In addition, several novel mouse strains were also generated. Vγ1.1Vδ6.3 TCR transgenic mice were generated using TCR isolated from Id3+/+ and Id3-/- mice and crossed to the C57Bl/6 background. The Trav15d-1-dv6d-1 and Trav15-1-dv6-1 gene segment deletions, and the Tcra-Tcrd locus 430 kb deletion (TcraΔ430 allele) were generated using CRISPR/Cas9 targeting and crossed to the C57Bl/6 background. Deletions of the E-box containing region downstream of Trav15d-1-dv6d-1 were similarly generated in TcraΔ15-1/Δ15-1;Id3fl/fl;R26ZsG/ZsG mice.
Wild animals	Not applicable
Reporting on sex	Sex and age-matched mice were analyzed and reported as aggregate as no phenotypic link to sex was detected.
Field-collected samples	Not applicable
Ethics oversight	All experiments were performed under the auspices of protocols approved by the Institutional Animal Care and Use Committees (IACUCs) at Fox Chase Cancer Center and/or Duke University

Note that full information on the approval of the study protocol must also be provided in the manuscript.

Plants

Seed stocks	Not applicable
Novel plant genotypes	Not applicable
Authentication	Not applicable

ChIP-seq

Data deposition

☒ Confirm that both raw and final processed data have been deposited in a public database such as GEO.

☒ Confirm that you have deposited or provided access to graph files (e.g. BED files) for the called peaks.

Data access links<br>*May remain private before publication.*	The resulting ChIP-seq analysis was performed as described and has been deposited in GEO (GEO: GSE162292). (https://www.ncbi.nlm.nih.gov/geo/query/acc.cgi?acc=GSE162292)
Files in database submission	GSM4948541 DN3 Input<br>GSM4948542 DN3 HEB IP<br>GSM4948543 DN3 E2A IP<br>GSM4948544 CD73- Input<br>GSM4948545 CD73- HEB IP

	GSM4948546 CD73- E2A IP<br>GSM4948547 CD73+ Input<br>GSM4948548 CD73+ HEB IP<br>GSM4948549 CD73+ E2A IP<br>GSM4948550 CD73 Negative Replicate 1<br>GSM4948551 CD73 Negative Replicate 2<br>GSM4948552 CD73 Positive Replicate1<br>GSM4948553 CD73 Positive Replicate2<br>GSM4948554 DN3 Replicate1<br>GSM4948555 DN3 Replicate2
Genome browser session (e.g. UCSC)	Not applicable

Methodology

Replicates	ChIPSeq data was generated from two independent sequencing runs, generated from two separate immunoprecipitations from cell populations generated in two independent cell culture experiments.
Sequencing depth	Sequencing depth for E protein ChIP-Seq ranged between 27 and 190 million reads, with an alignment rate ranging from 85-96.
Antibodies	Anti-HEB and anti-E2A affinity-purified polyclonal rabbit sera
Peak calling parameters	bowtie short read aligner was used to align ChIPSeq data. Aligned reads were subsequently analyzed using HOMER.
Data quality	Peaks are identified that are greater than 2-fold the peak width apart from one another with a false discovery rate of 0.001. All called peaks have 4-fold more normalized tags compared to the control (input/IgG) sample.
Software	ChIPSeq data was collected using a HiSeq2500. Reads were subsequently analyzed using bowtie and HOMER.

Flow Cytometry

Plots

Confirm that:

☒ The axis labels state the marker and fluorochrome used (e.g. CD4-FITC).

☒ The axis scales are clearly visible. Include numbers along axes only for bottom left plot of group (a 'group' is an analysis of identical markers).

☒ All plots are contour plots with outliers or pseudocolor plots.

☒ A numerical value for number of cells or percentage (with statistics) is provided.

Methodology

Sample preparation	Cells were harvested from fetal or adult thymus and spleen. Organs were mechanically disrupted and filtered through a 100um mesh strainer. Cells were counted, stained, and analyzed by flow cytometry.
Instrument	FACS data was collected using either a BD Fortessa or Symphony flow cytometer.
Software	FlowJo Software (v10,8,2) was used to analyze flow cytometry data.
Cell population abundance	Purities of sorted samples were over 95%.
Gating strategy	All gating schema begin with FSC-H v FSC-A for doublet discrimination, followed by FSC-H v SSC, and then a vital dye (PI, 7-AAD, etc).

☒ Tick this box to confirm that a figure exemplifying the gating strategy is provided in the Supplementary Information.

Magnetic resonance imaging

Experimental design

Design type	Not applicable
Design specifications	Not applicable
Behavioral performance measures	Not applicable

Acquisition

Imaging type(s)	Not applicable
Field strength	Not applicable
Sequence & imaging parameters	Not applicable
Area of acquisition	Not applicable

Diffusion MRI ☐ Used ☒ Not used

Preprocessing

Preprocessing software	Not applicable
Normalization	Not applicable
Normalization template	Not applicable
Noise and artifact removal	Not applicable
Volume censoring	Not applicable

Statistical modeling & inference

Model type and settings	Not applicable
Effect(s) tested	Not applicable

Specify type of analysis: ☐ Whole brain ☐ ROI-based ☐ Both

Statistic type for inference (See Eklund et al. 2016)	Not applicable
Correction	Not applicable

Models & analysis

n/a	Involved in the study
☒	☐ Functional and/or effective connectivity
☒	☐ Graph analysis
☒	☐ Multivariate modeling or predictive analysis
