# Supplementary material for: E proteins control the development of NKγδT cells through their invariant T cell receptor
Source: Nat Commun. 2024 Jun 13;15:5078. doi: 10.1038/s41467-024-49496-3 (PMC11176164; doi:10.1038/s41467-024-49496-3)
Supplement: Supplementary file 4 — Description of Additional Supplementary Files [file 41467_2024_49496_MOESM4_ESM.pdf]

# <u>Description of Additional Supplementary Files</u>

**Supplementary Data 1:** CDR3 sequences derived from single cell sequencing of the TCR complexes of the NKgdT cells in Id3+/+ and Id3-/- mice
